# Effect of electronic patient record use on mortality in End Stage Renal Disease, a model chronic disease: retrospective analysis of 9 years of prospectively collected data

**DOI:** 10.1186/1472-6947-7-38

**Published:** 2007-11-28

**Authors:** Victor E Pollak, Jonathan A Lorch

**Affiliations:** 1 MIQS Inc., 2100 Central Avenue, Suite 201, Boulder, Colorado 80301, USA; 2 The Rogosin Institute, 505 East 70th Street, New York, New York 10021, USA

## Abstract

**Background:**

In chronic disease, health information technology promises but has yet to demonstrate improved outcomes and decreased costs. The main aim of the study was to determine the effects on mortality and cost of an electronic patient record used in daily patient care in a model chronic disease, End Stage Renal Disease, treated by chronic maintenance hemodialysis. Dialysis treatment is highly regulated, and near uniform in treatment modalities and drugs used.

**Methods:**

The particular electronic patient record, patient-centered and extensively coded, was used first in patient care in 3 dialysis units in New York, NY in 1998, 1999, and 2000. All data were stored "live"; none were archived. By December 31, 2006, the patients had been treated by maintenance hemodialysis for a total of 3924 years. A retrospective analysis was made using query tools embedded in the software. The United States Renal Data System dialysis population served as controls. In all there were 1790 patients, with many underlying primary diseases and multiple comorbid conditions affecting many organ systems. Year by year mortality, hospital admissions, and staffing were analyzed, and the data were compared with national data compiled by the United States Renal Data System.

**Results:**

Analyzed by calendar year after electronic patient record implementation, mortality decreased strikingly. In years 3–9 mortality was lower than in years 1–2 by 23%, 48%, and 34% in the 3 units, and was 37%, 37%, and 35% less than that reported by the United States Renal Data System. Clinical staffing was 25% fewer per 100 patients than the national average, thereby lowering costs.

**Conclusion:**

To our knowledge, this is the first demonstration that an electronic patient record, albeit of particular design, can have a favorable effect on outcomes and cost in chronic disease. That the population studied has many underlying diseases affecting all organ systems suggests that the electronic patient record design may enable application to many fields of medical practice.

## Background

Care of chronic disease patients now predominates in medical practice, and accounts for >75% of US $2.1 trillion medical care costs [1]. Health information technology is believed essential in improving outcomes and decreasing costs [2,3]. Although an integrated electronic medical record, performance measures, and active performance monitoring has been associated with improved quality measures [4-6], hopes for effects on healthcare quality, efficiency, and costs have yet to be realized. One recent systematic review concluded that the evidence was sparse [7], another found little evidence that computerized clinical decision support has effected patient outcomes [8]. We found no literature on effects of electronic medical records on mortality in any chronic disease.

Over 30 years ago Fries concluded that in chronic disease: "a major failure of the traditional chart is its inability to indicate adequately complex temporal relationships between clinical, laboratory, and therapeutic events [9]." To address these complex temporal relationships, paper spreadsheets were used to manage clinical, laboratory, histological, and therapeutic details sequentially in renal involvement in systemic lupus erythematosus [10]. This approach was expanded to develop a new comprehensive patient-centered paper record to facilitate understanding of systemic and renal manifestations of diseases. After extensive testing it was converted to electronic form [11]. Discrete medical practice details for diseases affecting multiple organ systems were transformed into coded data, enabling rearrangement of data elements to facilitate clinical practice and observations over many years.

End Stage Renal Disease (ESRD) is complex, costly, and affects more than 500,000 patients in the United States [12]. As a model to test effects of this comprehensive, coded and analyzable, electronic patient-centered record (EPR) in chronic disease, ESRD has unique advantages. First, it is highly regulated, with frequent routine and *ad hoc *State and Federal oversight; treatment adequacy markers must be reported. Second, to start dialysis treatment, patients must meet mandated criteria. Third, treatments, devices, and drugs used are largely standardized. Fourth, dialysis procedures are also largely standardized and, for the most part, operator independent. ESRD often results from primary disease conditions such as diabetes mellitus, HIV/AIDS, multiple myeloma, systemic lupus erythematosus, arteriosclerosis, and hypertension; comorbid conditions affecting many organ systems are universal. Each patient requires lifetime care, receives 140–156 treatments and accumulates up to 11,600–21,000 individual data items yearly [13], is cared for in many locations by caregivers from several disciplines. Mandatory reporting ensures that the United States Renal Data System provides a national database and mortality standard with which to compare results. Moreover, 68–70% of US patients are treated in dialysis units owned by 5 (since recent mergers, 3) large dialysis "chains" [12]; each deploys an electronic medical record [14-17].

We here test the hypothesis that "Successful management and treatment of the patient and the important individual manifestations of a chronic disease require complex feedback systems that relate therapeutic interventions to clinical and laboratory information relevant to multi-organ systems over prolonged periods" [11]. We analyzed retrospectively data collected during patient care from 1998 onward using the EPR, enhanced continually since 1976 [13,18-21]. This study EPR was the test instrument. USRDS mortality data provided contemporaneous controls [12]. Favorable effects on mortality and cost were observed on ESRD patients treated by chronic maintenance hemodialysis (HD) for a total of 3,924 years over a 9-year period.

## Methods

### The setting

The study was done in 3 dialysis units managed by The Rogosin Institute (New York, NY), affiliated with New York Presbyterian Hospital and Weill Medical College of Cornell University. All provide treatment by in-center chronic HD. Unit A also trains and treats patients by peritoneal dialysis, home HD, and nocturnal self-HD. Eight full-time salaried Rogosin Institute staff nephrologists, who also care for renal and transplant patients, teach, and do clinical research, and 2 nurse practitioners care for patients in Unit A. In Unit B, there are 1 to 2 Rogosin nephrologists, 2 to 5 in private practice, and 1 nurse practitioner. In Unit C, the 3 nephrologists are in private practice.

### Participants

The patients were all 1790 patients treated by chronic maintenance HD from 91 days after ESRD start.

### Design of the study

The electronic patient record (Disease Manager Plus™, MIQS^® ^Inc, Boulder, CO) employs a relational database (Sybase^® ^Adaptive Server Enterprise, Sybase, Inc., Dublin, CA), running on a server computer (Sun^® ^Microsystems, Santa Clara, CA). It is accessed using a custom toolset (4D, Inc., San Jose, CA) from client personal computers in dialysis units, renal and transplantation practice, physician offices, hospital, and home. All clinical, administrative, and financial information is immediately accessible at all times on patients ever entered into the database. It serves all kidney disease care, including dialysis and transplantation. Subject to security considerations, lifetime patient data relevant to pertinent caregiver needs are accessed whenever and wherever needed. The security ensures confidentiality for clinical and financial information and for integrated electronic mail. Laboratory test results, radiology reports, pathology reports, and dialysis machine data download automatically into the database via MIQS-designed electronic interfaces. Dialysis machine data enable on-line chair-side and remote real-time monitoring including home nocturnal HD [21].

Coded data elements include diagnoses, procedures, symptoms, signs, medications, allergies, and hospitalizations. Patient-specific ICD-9-CM codes record reason(s) medications are prescribed and patients admitted to hospital. Notes are charted in free text or using templates. Advance directives, living will, do not resuscitate, treatment consent, and other documents are stored in the database and readily accessible. HD treatment screens record all details. HD orders and medications to be given during HD automatically populate treatment screen fields.

To provide clinically useful point-of-care reports embedded query tools are incorporated to organize data quickly in any way desired over any time period, to make knowledge available about individual patients, and groups. Reports that can be updated and organized at the point-of-care are user-designed to facilitate clinical decisions based on timely, complete, relevant, patient-specific, time-oriented data.

**Figure 1:**
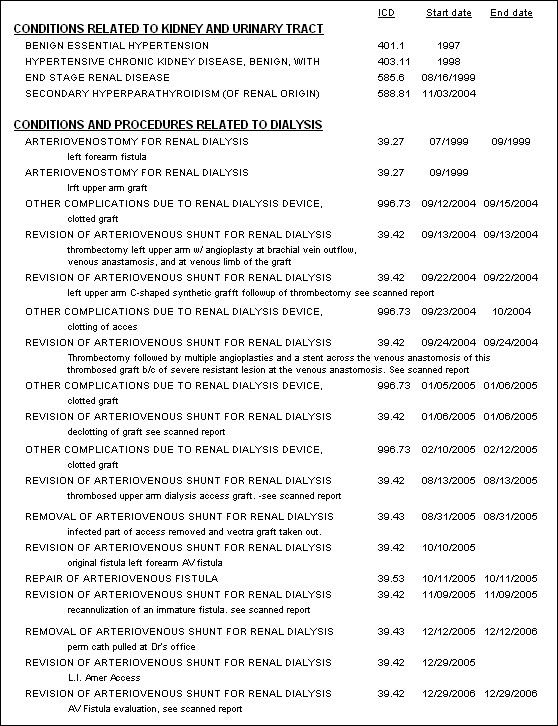
"Bird's eye view" of the first part of the lifetime history of a patient treated by in-center HD since August 1999. Disease conditions related to the kidney and urinary tract, and disease conditions and procedures related to dialysis are displayed. Organized for a dialysis patient, they are displayed chronologically under the corresponding headings. ICD-9-CM codes and descriptors, in upper case, are used to display the diagnoses and procedures. Brief comments, which had been added to the entry screens, appear in lower case beneath the ICD coded descriptors where they provide clinical color for the codes,

**Figure 2:**
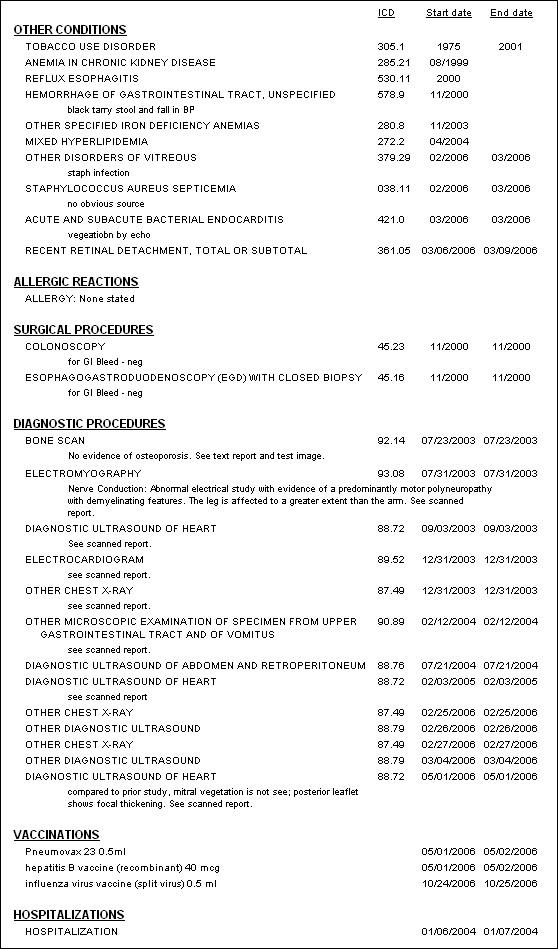
"Bird's eye view" of the second part of the lifetime history of a patient treated by in-center HD since August 1999. Disease conditions other than those related to kidney and urinary tract, allergic reactions, surgical procedures, diagnostic procedures, vaccinations, and hospitalizations are displayed. Reports of this type in which data are organized relevant to the needs of other fields such as cardiovascular disease, hematology, social work, etc are also immediately available.

**Figure 3:**
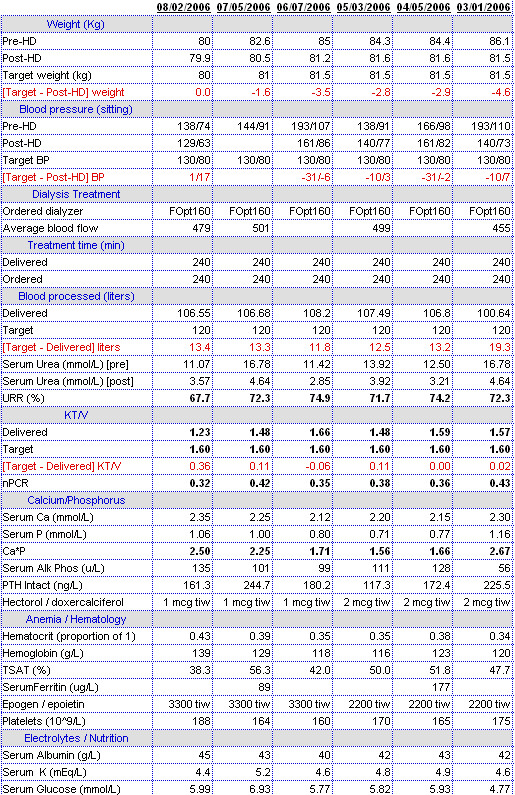
Report designed to enable caregivers to review the effects of treatment. It displays relevant "core" data of one patient for the present and the previous five months. Under headers weight, blood pressure, and dialysis treatment (with sub headers treatment time, blood processed, and Kt/V) the data derive from HD orders, HD treatments, and laboratory test results. They are arranged to provide feedback between orders for and treatments delivered. Relevant lab test results and medications are also displayed to enable review of common problems under headers calcium/phosphorus, anemia/hematology, and electrolytes/nutrition. Reports with an expanded relevant data set are immediately available for more detailed review of each of these problems.

The electronic starting point for patient encounters displays all relevant historical information including reports, medications, allergies, and patient-specific and rules based alerts and reminders. Encounters, tailored to specific functions such as HD, peritoneal dialysis, chronic kidney disease, and transplantation facilitate data entry and communication with others, e.g., referring physicians. Individual patient reports accessible on encounter and HD treatment screens include contemporaneous medications, comprehensive lifetime lists of diagnoses, surgical procedures, diagnostic procedures, allergies, adverse drug reactions, immunizations, and hospitalizations (Figures 1 and 2). Others display data over time in spreadsheet format from domains including signs, symptoms, medications, laboratory tests, HD orders and treatments, diagnoses, procedures, and hospitalizations (Figure 3).

Patient treatment groups, a special software functionality, define patients receiving treatment courses by in-center HD, other dialysis modalities, kidney and pancreas transplants. An in-center HD group is defined by dates of first and last HD in a treatment course. Reason the course ended is charted from a coded list that includes patient expired, recovered renal function, transferred to another dialysis unit, transferred to another group, e.g., kidney transplant.

### Data Analysis

Reports that incorporate information from multiple information domains were used for data analysis on patient groups. An integrated patient selection query tool enabled selection of cohorts for inclusion in reports. Among selection criteria were demographic elements, locations, alive or expired within a defined time range, presence or absence of ≤3 patient groups and ≤3 ICD coded diseases and procedures.

The Treatment History with Adjusted Dates report enabled much analysis. It adjusted dates automatically to first and last days of the chosen period, calculated days in the group, and displayed why the group ended. For the present analysis it was modified by adding age (at treatment history start), ethnicity, gender, ESRD date (first ever treatment by dialysis or transplantation), date of death, primary cause of ESRD. ESRD vintage was calculated as (Start date of dialysis in the period of study – ESRD date).

The report was generated using the patient selection query tool to select the pertinent HD cohort, Unit A, B, or C, individual calendar year or full 6–9 year spans. Days in the group were summed and average age calculated. Saved as an ASCII file, it was imported for further analysis into Microsoft^® ^Excel^® ^or SAS^® ^JMP™.

USRDS reports data only from the 91^st ^day after start of the first dialysis treatment [12]. To enable comparable analyses, data were sorted first by (Start date – ESRD date), and second by treatment group days. Patients starting dialysis within <91 days and with <91 dialysis treatment days were excluded. Those starting dialysis within 91 days of ESRD date were included if treated for >90 days; treatment group days were reduced by days prior to the 91^st^, as in USRDS.

The reason treatment was discontinued was examined, using the following conventions:

• Kidney transplants: Patients were deemed alive at the end of the prior dialysis course.

• Deaths: Date treatment was discontinued because patient expired was checked with date of death recorded under patient demographics. Known death within 30 days of transfer to hospital, nursing home, hospice or another dialysis unit was ascribed to the previous modality.

• Transfers to other units for continuing dialysis care: The database was searched for site of future dialysis care, and latest recorded patient-caregiver contact. Time from transfer to last documented contact was calculated. Patients documented alive ≥ 6 months after transfer were treated for analysis as alive at relevant study year-end. Patients with no such information available were considered "lost to follow up"

### Calculation of Mortality

Mortality per 1000 patient years was calculated for each individual study unit in each calendar year, as is done by USRDS, by dividing the number of deaths in each individual year by the total time in years that the patients were treated by HD in that calendar year, and multiplying by 1000.

### Data Verification

Analyses were made on several occasions. Dubious or missing values were checked and corrected as necessary in the EPR. Many coded values were missing because data had been charted as text only; coded values were entered from physician text notes. For final analysis, reports were run by individual calendar years and the entire 7–9 year period.

### Study Approval

The study was approved by the Institutional Review Board of Weill Medical College of Cornell University.

## Results

### Patients

The subjects of this report are 1790 patients treated by chronic maintenance hemodialysis from 91 days after ESRD start. Their mean age of 59.2 ± 16.16 (SD) years was 2.1 years older than that of USRDS (Table 1). There were more females, fewer Whites, more Blacks, more other/unknown racial groups, and more Hispanics. The proportion with ESRD due to diabetes mellitus type I was lower, whereas that due to diabetes mellitus type II, hypertension, glomerulonephritis, and polycystic kidney disease reflected national data (Table 2).

**Table 1:** Selected patient demographic data, at first HD treatment, between January 1 1998 and December 31, 2006

	All Units	USRDS*
Number of patients	1790	709,259
Mean age at start of first HD treatment during the study period (years)	59.2	57.1
Gender (male:female) (%)	52.9:47.1	54.7:45.3
Race (White: Black: Other/Unknown) (%)	42.4:36.4:21.2	63.3:28.5:8.2
Hispanic (%)	17.4	12.5

*USRDS data are prevalence of all ESRD patients for the years 1998–2005 combined.

**Table 2:** Primary disease causing ESRD

	All Units (%)	USRDS* (%)
Diabetes mellitus Type 1 (juvenile)	4.0	6.3
Diabetes mellitus Type 1I (adult onset or unspecified)	32.4	29.5
Glomerulonephritis	14.6	15.7
Hypertension	24.2	22.3
Systemic lupus erythematosus	1.9	1.9
Polycystic kidney disease	4.2	4.2
AIDS nephropathy	1.9	0.6
Neoplasm (renal and urological), myeloma, amyloidosis	1.6	0.9
Hydronephrosis, obstruction, infection	2.9	4.6

*USRDS data are prevalence of all ESRD patients for the years 1998–2005 combined.

Patients in the 3 Units differed in several characteristics. They were younger in Unit A than in B and C (Table 3). In Unit C more started treatment within 91 days of ESRD than in A and B, fewer within >2 years. In Unit A there were more with diabetes mellitus type I, glomerulonephritis, polycystic kidney disease, and fewer with diabetes mellitus type II (Table 4). There were fewer with hypertension in Unit C.

**Table 3:** Selected patient demographic data, at first HD treatment, in each dialysis unit

	Unit A (1998–2006)	Unit B (1999–2006)	Unit C (2000–2006)
Number of patients	858	515	417
Age at start of first HD treatment during the study period (years)	56.9	60.4	62.5
Gender (male: female) (%)	53.7:46.3	52.6:47.4	51.8:48.2
Race (White: Black: Other/Unknown) (%)	40.9:38.2:20.9	49.1:23.1:27.8	37.4:49.2:13.6
Hispanic (%)	19.1	17.8	13.4
Dialysis vintage ≤ 91 days (%)	41.2	41.9	65.0
Dialysis vintage > 2 years (%)	40.6	33.2	17.0
Dialysis vintage > 5 years (%)	23.9	16.3	4.7

**Table 4:** Primary disease causing ESRD in each dialysis unit

	Unit A (%)	Unit B (%)	Unit C (%)
Diabetes mellitus Type 1	5.7	2.3	2.4
Diabetes mellitus Type 1I	21.5	40.5	45.0
Glomerulonephritis	20.6	10.5	7.2
Hypertension	26.1	26.2	18.0
Systemic lupus erythematosus	2.2	1.4	2.1
Polycystic kidney disease	5.7	3.3	2.2
AIDS nephropathy	1.6	2.1	2.2
Neoplasms, myeloma, amyloidosis	1.9	1.0	1.9
Hydronephrosis, obstruction, infection	3.5	1.8	2.9

### Mortality

Over the entire study period, 13 patients recovered sufficient function to discontinue dialysis (Table 5). In Unit A, 12.7% of patients received a kidney transplant, 7.7% and 9.1% respectively in Units B and C. More Unit C patients (19%) were lost to follow up than A (11%) and B (8%) patients. In Units A, B, and C mortality was respectively 156, 171, and 173 per 1000 patient years, lower than the 1998–2005 USRDS HD mortality (229–241 per 1000 patient years) [12].

**Table 5:** Summary of patient outcomes in each dialysis unit over the 7–9 year period

	Unit A (1998–2006)	Unit B (1999–2006)	Unit C (2000–2006)
Years of observation	1926.7	1046.2	951.0
Recovered renal function	4	3	6
Received a kidney transplant	109	40	38
Followed in the database after transfer to peritoneal dialysis, home hemeral or nocturnal hemodialysis	41	3	3
Known to be alive after transfer to another dialysis facility	79	22	12
Lost to follow up after transfer to another dialysis facility	102	50	86
Deaths	303	178	164
Deaths per 1000 patient years	157.3	170.1	172.5

As might be expected when the number of patients in each unit was relatively small, mortality did vary substantially year-to-year (Table 6). Nevertheless, save for year 2001 in Unit A, mortality decreased strikingly in each Unit, from 2000 onward in Unit A, 2001 onward in B, and 2002 onward in C. Considered by year of EPR deployment, mortality was, with a single exception (Unit A, year 4), lower from year 3 onward.

**Table 6:** Patients treated, treatment duration, and mortality by calendar year in each dialysis unit. USRDS data are shown for comparison

Years	1998	1999	2000	2001	2002	2003	2004	2005	2006
Number of patients treated during each year									

Unit A	279	236	294	295	268	287	313	317	340
Unit B	NA	112	152	157	166	160	167	232	258
Unit C	NA	NA	154	171	171	180	190	192	193

Mean age (years) at start of HD treatment in each year									

Unit A	56.9	58.7	57.3	57.2	56.1	56.0	57.3	56.7	58.2
Unit B	NA	60.7	59.9	59.5	60.0	59.7	60.8	61.5	62.9
Unit C	NA	NA	62.5	63.4	64.2	61. 9	61.5	62. 6	62.5
									
USRDS*	56.2	56.5	56.8	57.0	57.3	57.5	57.7	57.9	NA

Duration of treatment (years)									

Unit A	193.5	173.8	221.1	208.1	207.9	219.4	232.8	235.3	244.8
Unit B	NA	81.0	100.2	118.6	121.6	121.9	130.6	169.3	202.9
Unit C	NA	NA	101.7	126.5	139.9	144.8	145.9	148.8	145.2

Number of deaths									

Unit A	36	35	38	47	29	19	38	34	27
Unit B	NA	25	26	17	21	21	14	27	27
Unit C	NA	NA	27	26	20	22	24	23	22

Mortality per 1000 patient years									

Unit A	186.1	201.4	171.8	225.8	139.4	86.2	163.2	144.5	110.8
Unit B	NA	308.7	259.5	143.4	172.7	172.3	107.2	159.5	133.1
Unit C	NA	NA	265.5	205.6	142.9	151.9	164.5	154.5	151.5
									
USRDS**	234	241	236	238	237	236	232	229	NA

* USRDS age data are mean age of all prevalent ESRD patients (both dialysis and transplant) in each calendar year.

** USRDS mortality data are for patients treated by HD.

**Figure 4:**
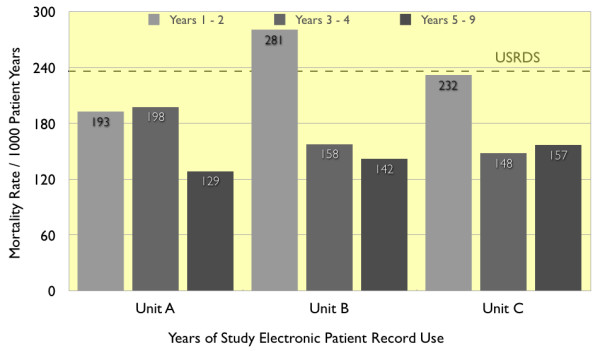
Mortality rate, by years of electronic patient record deployment, in dialysis Units A, B, and C. The mortality rate is compared with that reported by USRDS (horizontal line).

The effect of year-to-year variation may be expected to be less when the results are recapitulated in periods of two or more successive years. Mortality for years 1–2, 3–4, and 5–9 of EPR deployment is summarized in Figure 4. In Unit A, mortality in years 3–4 was 198 per 1000 years, similar to that in years 1–2. In years 5–9, mortality was 129 per 1000 years, a reduction of 35%. In Unit B, mortality was 44% lower in years 3–4 than in years 1–2, and 49% lower in years 5–8. In Unit C mortality was 36% lower in years 3–4 than in years 1–2, and 32% lower in years 5–7. By contrast, the contemporaneous USRDS mortality remained constant around 237 per 1000 years from 1998 to 2003, and decreased slightly to 232 and 229 per 1000 years in 2004 and 2005.

**Figure 5:**
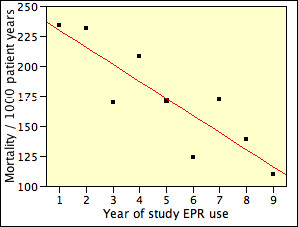
Data from all 3 dialysis units combined were used to analyze regression of mortality per 1000 patient years on years of study electronic patient record (EPR) use.

As a test of trend, the mortality data from all 3 Units were then combined and a simple regression analysis of mortality per 1000 years was calculated (Figure 5). This produced a regression of 244.4 – 14.18 per year of study EPR use (adjusted r^2 ^= 0.72, p = 0.0022). Thus, the mortality decreased by 28 per thousand every two years. It cannot be assumed, however, that this trend will continue with more extended use of the study EPR.

USRDS mortality calculations include deaths in hospitals, nursing homes, other dialysis units, and elsewhere. Complete knowledge of patients transferred is not available to individual dialysis units, nor was it for this analysis. Of 351 patients transferred to other units, 113 were documented alive an average of 2.32 years later. As some of the other 238 patients may have died and because death might have been attributed to Unit A, B, or C, mortality was recalculated, assuming that 25%, (approximating the USRDS 23.7%) died by end of the calendar year. So calculated, the difference in mortality between years 3–9 inclusive of electronic patient record deployment and years 1–2 changed little; it was lower by 17% in Unit A, 43% in Unit B, and 26% in Unit C.

**Figure 6:**
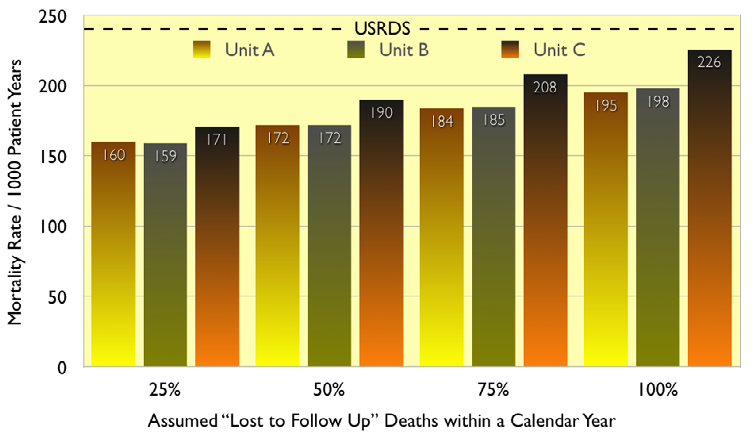
Mortality in years 3–9 combined, with varying assumptions about mortality within a calendar year among patients "lost to follow up".

Mortality in years 3–9 inclusive was recalculated for the 3 Units combined assuming that 25%, 50%, 75%, or 100% of those lost to follow up died by end of the calendar year in which they were transferred (Figure 6). Mortality would have been 31%, 26%, 20%, and 14% less than USRDS assuming respectively that 25%, 50%, 75%, or 100% of those lost to follow up died.

### Hospital Admissions

Over the entire study period, patients in Units A and B were admitted to hospital an average of 1.24 (range 1.08 to 1.39) and 1.32 (range 1.01 to 1.65) times per year, i.e., 39% and 35% less frequently than the USRDS rate of 2.04 [12]. Patients in Unit C were admitted to hospital 2.97 times per year in years 1–2 of EPR deployment, a rate reduced to 2.01 in years 3–7.

### Staff

Since 2004 patient and staff counts as of December 31 of each year have been provided in the annual Dialysis Facility report by the ESRD Network of New York. Patients treated by both HD and peritoneal dialysis are included. In 2004 and 2005 clinical staff numbered 14.1, 13.4, and 13.5 per 100 patients in Units A, B, and C respectively. In those two years the national average was 18.41 clinical staff per 100 dialysis patients [12]. The data for the three study Units were combined to compare with the USRDS data (Table 7). Clinical staff in the study Units numbered 13.79 per 100 patients, i.e., 25.1% fewer than the national average. Nurses, patient care technicians, dietitians, and social workers were respectively 29.1%, 24.7%, 18.6% and 8.5% fewer per 100 patients than the USRDS.

**Table 7:** Counts of patients and patient care staff on December 31, 2004 and December 31, 2005

	Patient care staff		Staff per 100 Patients	
	Study Units*	USRDS	Study Units	USRDS

Patients**				

	624	326, 574	NA	NA

Staff (Full-time equivalents)***				

Total patient care staff	86	60,112	13.79	18.41
Nurses (RNs and LPNs)	34	25,109	5.45	7.69
Patient care technicians	39.5	27,466	6.33	8.41
Dietitians	5.75	3,675	0.92	1.13
Social workers	6.75	3,863	1.08	1.18

* Data are means of counts at the end of years 2004 and 2005.

** Patients treated by hemodialysis and by peritoneal dialysis.

*** Staff who care for hemodialysis and peritoneal dialysis patients. One part-time employee is considered equivalent to 0.5 full-time employee.

## Discussion

Using an earlier EPR version in a single dialysis unit, we reported mortality 25% below the 1980–1989 national average [13,22]. Both EPR software and the national database have been greatly enhanced since. The present study is the first demonstration, to our knowledge, that use of an electronic patient record in any chronic disease practice favorably effects mortality.

Stead wrote recently that improvements from information technology implementation "are difficult to quantify in a practice while changing people's roles, process, and technology at the same time. Most measures have an immediate impact on process whereas many of the expected benefits are in long-term clinical outcomes [8]." We had no *a priori *knowledge of time needed from initial EPR implementation to development of a measurable outcome, particularly when dependent on many underlying diseases, comorbid factors, care, and other variables. A favorable effect by year 3 seems reasonable because staff had to learn to use and become comfortable with the technology while continuing daily patient care activities, and lower mortality in year 3 is likely to reflect improved quality of care in year 1 and/or 2. This was clearly evident in Units B and C by year 3, and in Unit A by year 5. Lower mortality was observed consistently thereafter for 5 years in Unit A, 6 years in Unit B, and 5 years in Unit C.

That mortality of the study population was less than USRDS might be explained by differences between demographic and comorbid factors of the study and USRDS populations. To test for this possibility we obtained standardized mortality ratios (SMR) generated by the University of Michigan Kidney Epidemiology and Cost Center [23]. Since 2001, SMR has been calculated from a Cox model, adjusting for age, race, ethnicity, gender, diabetes, ESRD duration, patient comorbidities and body mass index at incidence, and population death rates. SMR trends in 2001–2006 were similar to those in our analysis. Compared on a year-by-year basis, SMR and the study patient mortality correlated significantly (adjusted r^2 ^= 0.30, p = 0.011), suggesting that the decreased mortality in 2001–2006 was not due to differences in demographic and comorbid factors of the study and USRDS populations. The demographic and comorbid factors of the population of each Unit, which varied year-to-year almost imperceptibly, cannot explain the lower mortality observed in each Unit. This supports the view that the lower mortality when compared with USRDS was not due simply to a center effect.

We sought to identify other factors such as change in management patterns or dialysis technology that might have contributed to improved outcomes; no obvious ones were found. Increased number and/or professional qualification of patient care staff is one possible explanation; this was not the case. As in an earlier report [13], there was a favorable effect on staff efficiency (Table 7). Compared with years 1–2 hemoglobin and serum albumin, indicators of patient well being, changed little: hemoglobin increased 5% in years 3–9; serum albumin and serum iron were unchanged. Kt/V, a widely used index of dialysis adequacy, increased in years 3–9 by 11%, 2%, and 4% in Units A, B, and C respectively. Mortality decreased most in Units B and C, where the change in the index was very small.

## Conclusion

How is it possible that the particular study EPR can have played so important a role? Two properties of the EPR are crucial to understanding: it is patient-centered, and it is extensively coded.

The patient-centered study EPR captures, stores, and retrieves on-line and without delay all patient-specific medical data from multiple information domains including diagnoses, procedures, symptoms, signs, medications, orders, test results, and dialysis treatments [11,18]. It does so for all venues of care and for care provided by any caregiver, especially important in dialysis where all patients have multiple systemic diseases that need repeated evaluation and treatment by multiple providers over many years at a variety of sites. Unlike disease registries that usually focus on a single disease entity and its assumed complications, the study EPR is a generalized model of medicine that makes no assumption about future co-morbidities, complications, or outcomes in its data model. On the contrary it captures, stores, and retrieves any or all that might occur.

Extensive coding of the study EPR is essential to its utility. The ultimate products of virtually all electronic medical records are notes similar to those in a paper chart; data cannot be added or rearranged to explore varied and unexpected facets of the patient's condition(s), nor can they be viewed over time. The products of the study EPR are reports that contain many domains of data appropriate to a particular disease at any particular moment in time in care of the patient (Figures 1, 2, and 3). Their function is not static documentation of a moment; rather it is to display what has occurred over time, to evaluate and/or change the intervention, and then repeat the process. Essentially the report facilitates continuous quality improvement at the point-of-care [24].

**Figure 7:**
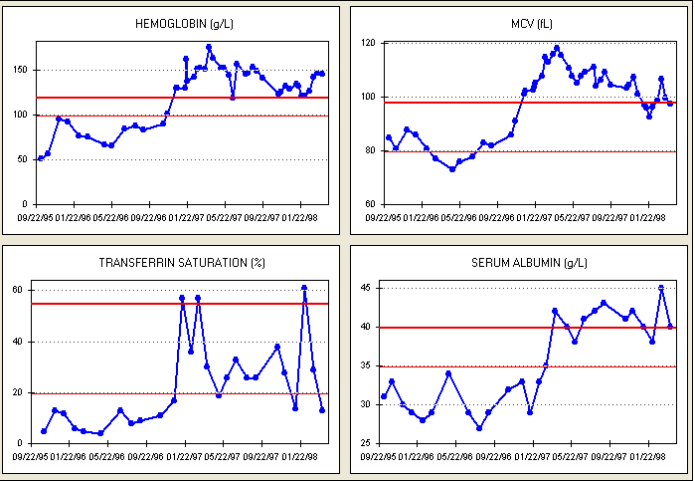
First treated by HD in January 1994, this 39 year old diabetic patient was transferred to a dialysis unit using the study EPR in November 1995. He received intravenous (IV) iron in three 1 g courses between November 1995 and June 1996. A modest improvement in the hemoglobin level occurred, but transferrin saturation (TSAT, an important measure of iron deficiency), mean corpuscular volume (MCV), and serum albumin level (a predictor of mortality) remained low. Recognizing that conventional IV iron replacement had been ineffective, 1 g courses of IV iron were again given starting in November 1996. With appropriate feedback, the courses were repeated whenever measures of iron deficiency were low. Not only was there an excellent hemoglobin response, but two unexpected findings were apparent: MCV increased to well above the normal range, and serum albumin increased to a range predictive of relatively low mortality. These observations were then confirmed in a large patient cohort [26].

**Figure 8:**
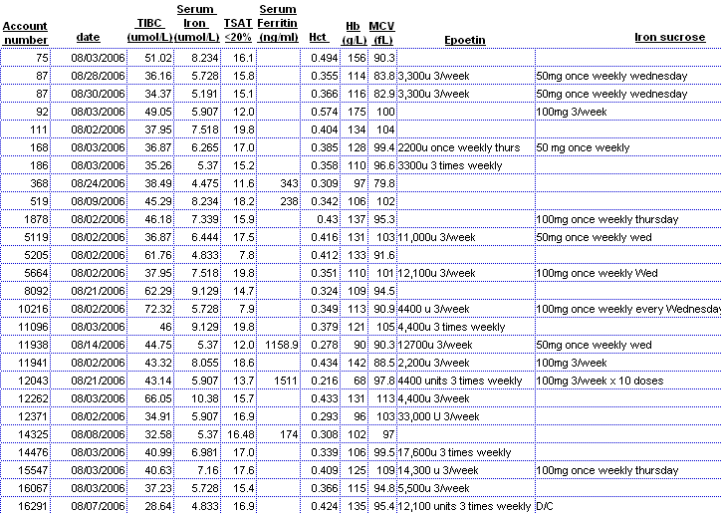
Iron deficiency and the need for iron replacement treatment are common problems in patients treated by HD. TSAT is an important marker for iron deficiency. This multi-patient report, run each month, displays relevant laboratory data and medications on patients selected for display because the TSAT level was low (<20%, column 5). It enables attention of caregivers to those patients (approximately 60 of 300) in whom review of need for iron replacement is particularly desirable.

**Figure 9:**
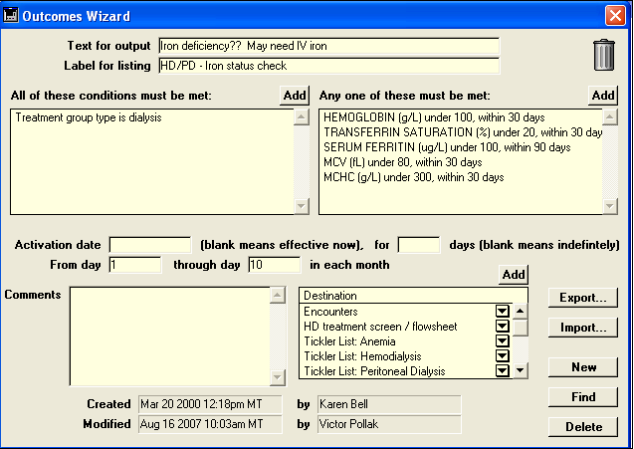
Rules based alert to the possible need for IV iron in patients treated by dialysis. Low values for any one of five laboratory measurements may trigger the alert, which is activated for the period when laboratory measurements are obtained (first 10 days of each month). The alert is directed to appear on encounter screens, HD treatment screens, and in several tickler reports.

These properties of the study EPR made possible:

(1) Computerized data entry with, and generation of, clinically relevant reports thereby eliminating communication and process errors.

(2) All information needed for skilled clinicians to make best judgments about patient care was always available immediately in clinical reports. Data in reports were complete, from multiple domains, and were viewed sequentially over time (Figure 3). We believe that complete, accurate, up-to-date, and focused time-oriented information had a favorable effect on work flow, facilitated understanding of the complex interrelations that effect outcome, enabled clinicians to make best management decisions, and therefore favorably impacted survival.

(3) Aberrant findings in one or a few patients were easily recognized (Figure 7), and their prevalence investigated rapidly [25,26]. This knowledge led to development of reports (Figure 8) and rules based alerts and reminders (Figure 9) for use in daily clinical practice. Knowledge so derived also enabled survival analysis [27], generated new understanding of a disease [25], decreased symptoms, improved well being [13,18,22], and detected and solved unexpected problems [19].

(4) Development of protocols and their timely updating as new data became available. Recognition of a high prevalence of iron deficiency in anemic epoietin treated dialysis patients led to systematic study, with repeated feedback, of intravenous iron repletion. Unexpected findings included: a very significant rise in serum albumin – an excellent outcome marker [28] – and in indirectly measured muscle mass; iron deficiency without iron deficiency anemia; decreased hospital admissions and stay [26]. This knowledge was disseminated in the study units when one of us (JAL) joined the Rogosin staff.

A trial of the study EPR with a randomly selected control group has the potential to provide evidence of cause and effect but would be difficult to design and control, and expensive to carry out for the many years needed. The present study compares data between groups and across time periods and uses two national standards, USRDS data [12] and standardized mortality ratio data [23], for contemporaneous controls. Unadjusted and adjusted mortality decreased comparably in 3 practice environments using the study EPR. Nationwide, where 70% of patients are treated by 3–5 corporations that use a traditional electronic medical record focused on management and billing, there was no concurrent change. With the limitations inherent in an observational study, the data provide evidence that benefits in long-term clinical outcomes can be achieved with the study EPR although cause and effect cannot be proven.

The study EPR had a favorable effect on mortality in ESRD patients treated by dialysis. Are similar results possible in other chronic diseases, and with other medical information systems? The challenges are great, as reviewed recently in a study of practice guidelines and quality of care for older patients with multiple comorbid conditions [29]. In 1977 we wrote, "At any time in the course of an illness, the patient may develop one, or many of the universe of symptoms, physical signs, and laboratory abnormalities that occur in clinical medicine as a whole" [11]. The study EPR database, and its integrated query tools, were designed to enable deployment in many fields of medicine and chronic diseases. If this and other EPR databases are to yield comparable results, we believe it essential that they adhere closely to the dictum that the medical record must be able to indicate complex temporal relationships between clinical, laboratory, and therapeutic events [9], and in both individuals and groups of patients.

## Competing interests

Victor E. Pollak is Founder, Member of the Board of Directors, owner of stock, and full-time employee of MIQS, Inc.

Jonathan A. Lorch is owner of stock of MIQS, Inc.

## Authors' contributions

VEP designed the medical content of the database, and implemented it on paper, 1968–75. In collaboration with software engineers, implemented 3 generations of the EPR starting in 1976, 1982, and 1991 respectively; the third generation is the study EPR used in this manuscript. VEP was responsible for design and implementation of the data analysis. In collaboration with JAL, responsible for writing the manuscript.

JAL implemented the software for clinical, administrative, and billing functions in all clinical facilities of The Rogosin Institute, including the 3 dialysis units that are the subjects of the study. In collaboration with VEP, responsible for writing the manuscript.

Both authors read and approved the final manuscript.

## Pre-publication history

The pre-publication history for this paper can be accessed here:


